# Early Vascular Aging in Children With Tuberous Sclerosis Complex

**DOI:** 10.3389/fped.2021.767394

**Published:** 2021-11-29

**Authors:** Piotr Skrzypczyk, Anna Maria Wabik, Michał Szyszka, Sergiusz Józwiak, Przemysław Bombiński, Aleksandra Jakimów-Kostrzewa, Michał Brzewski, Małgorzata Pańczyk-Tomaszewska

**Affiliations:** ^1^Department of Pediatrics and Nephrology, Medical University of Warsaw, Warsaw, Poland; ^2^Department of Pediatrics and Nephrology, Doctoral School, Medical University of Warsaw, Warsaw, Poland; ^3^Department of Pediatric Neurology, Medical University of Warsaw, Warsaw, Poland; ^4^Department of Pediatric Radiology, Medical University of Warsaw, Warsaw, Poland

**Keywords:** tuberous sclerosis complex, early vascular aging, central blood pressure, arterial stiffness, common carotid artery intima-media thickness, arterial hypertension, children

## Abstract

**Objectives:** Experimental data indicate that activating mutations in the mTOR (mammalian target of rapamycin) pathway may lead to abnormal arterial wall structure. Vascular anomalies like arterial stenoses are reported in pediatric patients with tuberous sclerosis complex (TSC). In addition, large renal lesions (angiomyolipoma—AML and cysts) are risk factors for arterial hypertension in adult patients with TSC. This study aimed to assess blood pressure, including central blood pressure and arterial damage (early vascular aging—EVA) in children with TSC.

**Materials and Methods:** In a group of 33 pediatric patients with TSC (11.13 ± 4.03 years, 15 boys, 18 girls), we evaluated peripheral and central office blood pressure, 24-h ambulatory blood pressure, and arterial damage: aortic pulse wave velocity (aPWV) [m/s], [*Z-*score], augmentation index (AIx75HR [%]), common carotid artery intima-media thickness (cIMT) [mm], [*Z-*score], stiffness of common carotid artery (E-tracking), renal lesions in magnetic resonance and ultrasonography, and selected biochemical parameters. The control group consisted of 33 healthy children (11.23 ± 3.28 years, 15 boys, 18 girls).

**Results:** In TSC group 7 (21.2%) children had arterial hypertension, 27 (81.8%) children had renal angiomyolipomas, 26 (78.8%)—renal cysts, and 4 (12.1%) patients were treated with mTOR inhibitors (2 patients with everolimus and 2 patients with sirolimus) at the moment of evaluation. Children with TSC had higher central systolic blood pressure (AoSBP) (98.63 ± 9.65 vs. 90.45 ± 6.87 [mm Hg], *p* < 0.001), cIMT (0.42 ± 0.05 vs. 0.39 ± 0.03 [mm], *p* = 0.011), cIMT *Z-*score (0.81 ± 1.21 vs. 0.16 ± 0.57, *p* = 0.007), aPWV (4.78 ± 0.81 vs. 4.25 ± 0.56 [m/s], *p* = 0.003) and aPWV *Z-*score (−0.14 ± 1.15 vs. −0.96 ± 0.87, *p* = 0.002) compared to healthy children, without differences in AIx75HR (8.71 ± 15.90 vs. 5.24 ± 11.12 [%], *p* = 0.319) and stiffness of common carotid artery. In children with TSC AoSBP correlated positively with serum cystatin C concentration (*r* = 0.377, *p* = 0.030) and with maximum diameter of renal cyst (*R* = 0.419, *p* = 0.033); mean arterial pressure (MAP) 24 h *Z-*score correlated with serum cystatin C concentration (*R* = 0.433, *p* = 0.013); and aPWV *Z-*score with daily urinary albumin loss [mg/24 h] (*R* = 0.412, *p* = 0.029).

**Conclusions:** Children with tuberous sclerosis complex are at risk of elevated central blood pressure and early vascular aging. In children with TSC, blood pressure and arterial stiffness are related to renal involvement.

## Introduction

Tuberous sclerosis complex (TSC, Bournevile-Pringle disease) is an autosomal dominant disorder found in 1:5,800–1:12,500 births. At present, TSC is diagnosed on the basis of recently updated 2021 International Tuberous Sclerosis Complex Consensus Group (ITSCCG) genetic and clinical criteria (1). The mutation in tumor suppressor genes: *TSC1* (chromosome 9q34) or *TSC2* (chromosome 16p13) are found in 85–90% of the TSC patients; remaining individuals were found to have mutations in non-coding regions of the genes or to show genetic mosaicism (2). In most patients, family history is negative (*de novo* mutation); approximately 30% of cases are inherited from one of the affected parents (3). *TSC1* and *TSC2* genes encode natural inhibitors of the mTOR (mammalian target of rapamycin) signaling pathway, i.e., hamartin and tuberin, respectively. Constant activation of the mTOR pathway leads to uncontrolled cell proliferation and formation of hamartomas, benign neoplasms, and rarely, malignant neoplasms in virtually all parts of the body. Thus, the spectrum of TSC-associated disorders includes skin lesions (e.g., depigmented spots, facial angiofibromas, shagreen patches, seen in ~90% of patients), retinal lesions (87% of patients), central nervous system abnormalities (70–90% of patients), heart (self-limiting rhabdomyoma found in approximately half of the patients), lungs (angiomyolipoma and lymphangioleiomyomatosis present mainly in women) and, finally, diversified renal lesions (1).

Renal abnormalities are present in as many as 50–80% of TSC individuals and are the second cause of mortality (just after brain tumors) in these patients. The spectrum of renal abnormalities involves angiomyolipomas (AML)—present in 55–90% of patients with kidney lesions, renal cysts, glomerulocystic disease, oncocytoma, and renal cell carcinoma (RCC) (4, 5). Angiomyolipomas belong to a family of neoplasms called perivascular epithelioid cell tumors. They can be classified histologically as typical (triphasic or lipid-rich) or atypical or fat-poor (monophasic or epithelioid). Typical, lipid-rich AMLs are benign tumors histologically characterized by (in varying proportions) proliferation of spindle cells, epithelioid cells, and adipocytic cells in concert with many abnormal, thick-walled blood vessels (6). AMLs pose a risk of life-threatening spontaneous bleeding (Wünderlich syndrome) once the tumor's diameter exceeds 30 mm (1, 6).

Experimental data indicate that activating mutations in the mTOR pathway may lead to degenerative phenotype and uncontrolled proliferation of vascular smooth muscle cells (SMC) and abnormal structure of arterial wall (7, 8). Arterial stenoses (e.g., renal artery stenosis, mid-aortic syndrome) or aneurysms have been reported in children and adults with TSC (9–12). In addition, large renal lesions are risk factors for arterial hypertension in adult patients with TSC (3, 13). These factors might theoretically put children with TSC at risk of arterial damage and early vascular aging (EVA), a state of accelerated adverse changes in the biochemical and cellular components of the vascular tree (14, 15). However, to the best of our knowledge, there are no reports on arterial damage in both children and adults with TSC. Thus, this study aimed to assess whether children with TSC are at risk of EVA and evaluate potential arterial damage determinants in this group of patients.

## Materials and Methods

### Study Group

**Figure 1:**
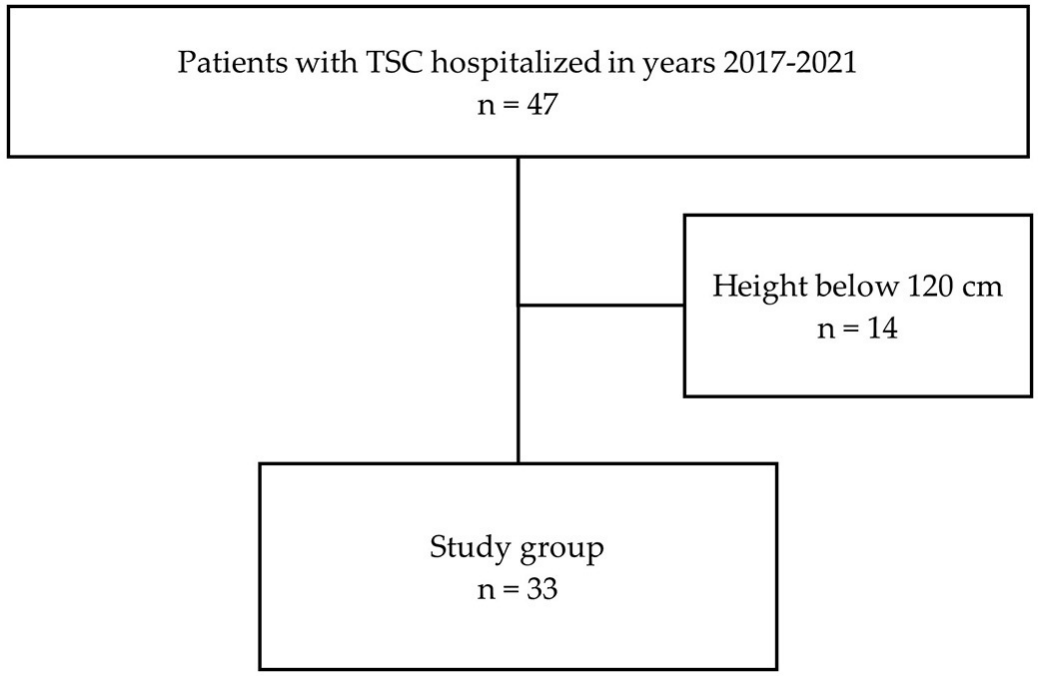
Flowchart of the patients' recruitment (TSC—tuberous sclerosis complex).

**Figure 2:**
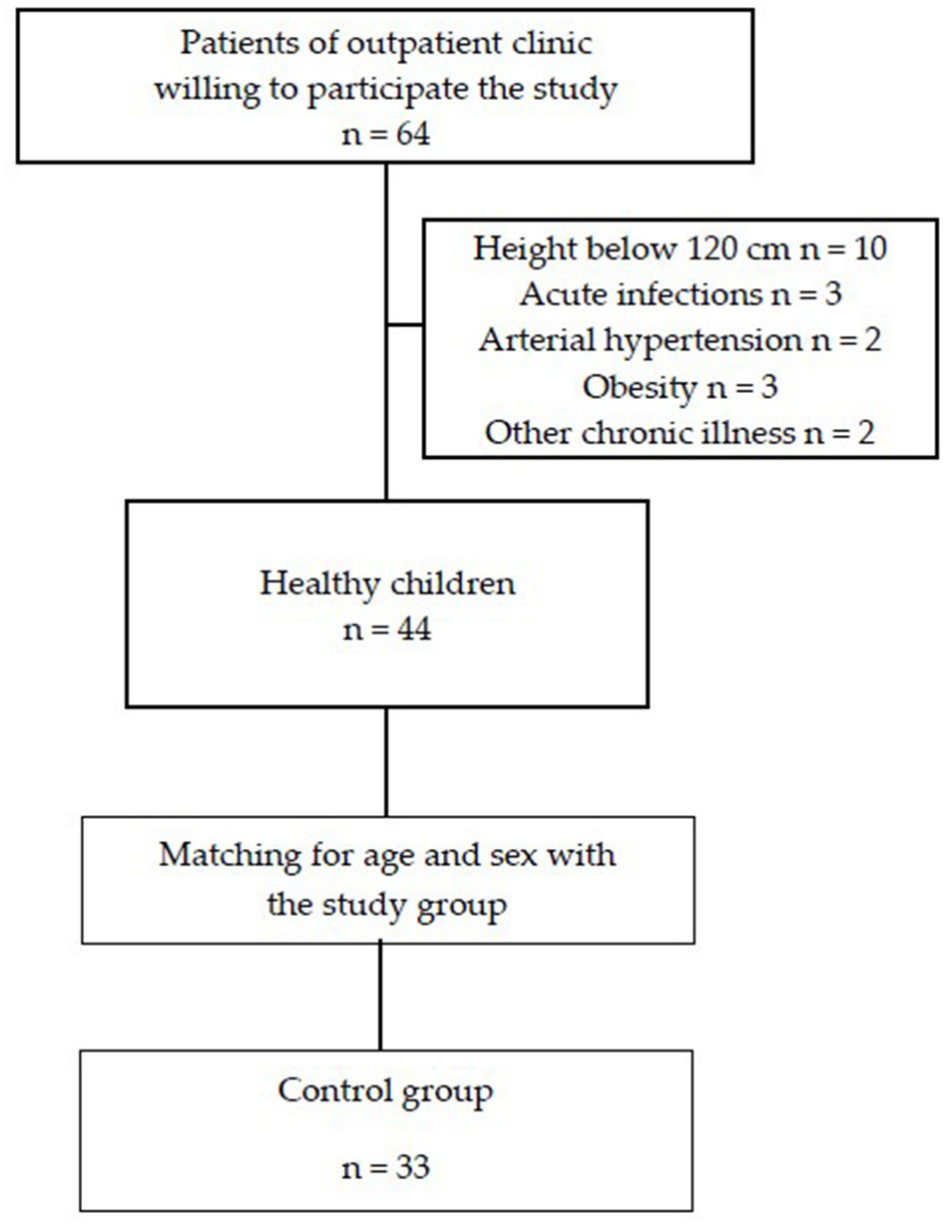
Flowchart of the control group recruitment.

This single-center cross-sectional study included patients with TSC treated in 2018–2021 in one tertiary center of pediatric nephrology. The criterion for inclusion in the study was confirmed TSC following ITSCCG recommendations (1, 16). The exclusion criteria were: height below 120 cm or acute infectious disease (temporary 2-week exclusion). Thirty-three age- and sex-matched healthy subjects were included in the control group. Healthy children were recruited from patients of outpatient University Hospital. Participation in the study was proposed with the following exclusion criteria: height below 120 cm, acute infectious disease, known chronic illness (including kidney, heart, and inflammatory disease), obesity, and arterial hypertension. The flowcharts of the study and control groups are presented in Figures 1, 2, respectively.

The authors obtained approval from the local Bioethical Committee before initiating the research (approval no. KB/145/2017, 4th July 2017). All procedures involving human participants were in accordance with the highest ethical standards of the institutional research committee and were performed according to the Declaration of Helsinki on the treatment of human subjects and its later amendments. Informed consent was obtained from all participants' representatives and participants (≥16 years) before enrolling in the study.

### Clinical and Biochemical Parameters

Based on individual medical records, we evaluated the following clinical parameters in all TSC individuals: family history of tuberous sclerosis complex, presence of TSC-related neurological symptoms: developmental delay and epilepsy, presence of arterial hypertension, and medications used, including mTOR inhibitors, antiepileptic and antihypertensive drugs. In all children with TSC, the following basic anthropometric parameters were assessed: age [years], sex, body height [cm], body weight [kg], and body mass index [kg/m^2^]. Anthropometric parameters were compared with the standards for the Polish population and presented in the form of a *Z-*score (17). According to WHO recommendations, overweight and obesity were defined as a BMI *Z-*score above 1 and 2, respectively.

All TSC patients had following biochemical parameters evaluated: serum concentrations of creatinine [mg/dL], urea [mg/dL], cystatin C [ng/mL], uric acid [mg/dL], total, HDL- and LDL-cholesterol [mg/dL], and triglyceride [mg/dL]. Also, daily urinary albumin loss was assessed in all the children. The estimated glomerular filtration rate (eGFR) was calculated in all patients [mL/min/1.73 m^2^] according to the revised 2009 Schwartz formula (18). Impaired renal function was defined as eGFR below 60 mL/min/1.73 m^2^ [which responses to chronic kidney disease 3G according to (19)], and hyperfiltration was diagnosed when eGFR was equal or exceeded 140 mL/min/1.73 m^2^ (20). Normal values of cholesterol and triglycerides were taken from the Expert Panel on Integrated Guidelines for Cardiovascular Health and Risk Reduction in Children and Adolescents; National Heart, Lung, and Blood Institute (21), and hyperuricemia was recognized when uric acid was ≥5.5 [mg/dL] (22). Elevated urinary albumin loss was defined as daily albuminuria >30 mg/24 h.

### Blood Pressure and Parameters of Arterial Damage

Blood pressure measurements were performed using the oscillometric method (Welch Allyn Patient Monitor, Welch Allyn, Skaneateles Falls, NY, USA) in line with ESH recommendations. They were analyzed using pediatric normative values ([mm Hg], *Z-*scores) (23, 24). According to the American Heart Association guidelines, all the TSC children also had 24 h ambulatory blood pressure measurement performed (Oscar 2 Suntech with Sphygmocor Inside, SunTech Medical Inc., Morrisville, NC, USA) (25). Systolic, diastolic, and mean blood pressures (SBP, DBP, MAP, respectively), blood pressure loads, and nighttime blood pressure dipping (DIP) were analyzed. DIP below 10% was considered as disturbed circadian rhythm (25). The blood pressure cuff was chosen following ESH recommendations and the devices' instructions (26).

The assessment for early vascular aging was performed using the following methods: an ultrasonographic examination of the common carotid artery (ALOKA Prosound Alpha 6, Hitachi Aloka Medical Ltd., Tokyo, Japan)—common carotid artery intima-media thickness (cIMT) [mm], *Z-*score (27), and common carotid artery local stiffness (E-tracking); applanation tonometry (Sphygmocor, ATCOR, Sydney, Australia)—central aortic blood pressure, pulse wave analysis, and aortic (carotid-femoral) pulse wave velocity (aPWV) [m/s], *Z-*score (28). All arterial measurements were performed by a single investigator (P.S.) in a quiet room with a controlled temperature (20 ± 5°C) after 5 min rest. cIMT was measured in all patients in a supine position using a manual method approximately 1 cm proximal to the carotid bulb on the distal carotid wall. Six cIMT measurements were obtained and averaged, three on the left and three on the right side. Peripheral pressure waveforms were recorded from the right radial artery at the wrist in a sitting position, and the transfer function was used to generate the central pressure waveform. aPWV was measured in a supine position and calculated as a difference in the carotid-to-femoral path length divided by the difference in R wave to the foot of the pressure wave taken from the superimposed ECG and pressure tracings. The path length was measured as the distance from the right carotid sampling site to the jugular notch, subtracted from the distance from the jugular notch to the right femoral sampling site (27). Peripheral pressure waveform and aPWV were obtained three times, and the mean value was analyzed.

### Evaluation of Renal Lesions

In all patients, abdominal ultrasonography was performed using a Philips Epiq 5G device (Royal Philips, Amsterdam, The Netherlands) in B-mode. Renal length [mm], echogenicity and corticomedullary differentiation, and the presence of renal parenchymal changes, including typical TSC lesions: angiomyolipoma (AML) and cysts were evaluated. The longest dimension of the largest lesion [mm] was assessed for AML and cysts. In patients diagnosed with arterial hypertension duplex Doppler ultrasonography was performed to exclude renal artery stenosis. In 24/33 (72.7%) patients, magnetic resonance imaging (MRI) of the abdomen was performed with a MAGNETOM Skyra 3T 3-tesla scanner (Siemens AG, Berlin, Germany) in T2-weighted, DWI, and T1-weighted sequences before and after intravenous administration of the contrast agent—Gadovist (gadobutrol) (Bayer AG, Leverkusen, Germany). Renal length [mm], renal cortical signal and differentiation, and presence of renal parenchymal lesions (angiomyolipomas, cysts) with the assessment of the largest dimension of the largest lesion [mm] were evaluated. Fat-poor AMLs were recognized according to the Polish Society of Nephrology recommendations (29). In case of discrepancy between MRI and ultrasound findings, MRI findings were considered conclusive.

### Statistical Analysis

The results were statistically analyzed using TIBCO Statistica 13.3 software (TIBCO Software Inc., Palo Alto, CA, USA). The normality of variables was studied using the Shapiro–Wilk test. The numerical data obtained were presented as mean and standard deviation (SD) (normally distributed data) or median and interquartile range (IQR, Q1–Q3) (non-normally distributed data). Normally distributed data were compared with Student *t*-test for independent groups and non-normally distributed data using the Mann–Whitney U test. The relationship between the two groups of variables was analyzed using Pearson correlation or Spearman rank correlation (depending on the distribution). Percentages in both groups were compared using the chi-square test. A *p*-value < 0.05 was considered statistically significant.

## Results

### Clinical and Biochemical Parameters of Children With Tuberous Sclerosis Complex

Clinical and biochemical parameters in the studied children were presented in Table 1. In the group of 33 children with TSC, there were 4 (12.1%) overweight patients and no obese children. Three (9.1%) patients had previously diagnosed arterial hypertension—all these patients were treated with angiotensin-converting enzyme inhibitors (2 with enalapril, 1 with ramipril). In addition, arterial hypertension was diagnosed at the moment of evaluation in 4 patients based on abnormal ambulatory blood pressure monitoring (including three masked hypertension patients). Most of the children had developmental delay and epilepsy. Four (12.1%) patients were treated with mTOR inhibitors. Three children received mTOR inhibitors due to large (>3 cm) renal angiomyolipomas (rapamycin in 2 and everolimus in 1), and one child was treated with everolimus due to the presence of non-operative SEGA tumors. Most patients had kidney involvement revealed in MRI or US. There were only 3 (9.1%) children without renal lesions. The largest AML (71 mm in maximal diameter) was found in a 17-year-old female patient who started rapamycin treatment. Two male patients aged 7.7 and 9.3 years had genetically confirmed contagious genes syndrome (deletion involving TSC2 and PKD1 genes) with very large renal cysts (maximal cyst diameter −35 and 44 mm, respectively). Impaired renal function was found in none of the patients, but in 11 (33.3%) children, hyperfiltration was revealed. Hyperuricemia was found in 6 (18.2%) children with the highest uric acid concentration 6.3 mg/dL. Acceptable total cholesterol (<170 mg/dL) was found in 26 (78.8%), borderline high total cholesterol (170–199 mg/dL) in 5 (15.2%) and high (≥200 mg/dL) in 2 (6.0%) children; acceptable triglycerides (<75 mg/dL in 0–9 years and <90 mg/dl in 10–19 years) was revealed in 24 (72.7%), borderline high triglycerides (75–99 mg/dL in 0–9 years and 90–129 mg/dl in 10–19 years) in 5 (15.2%) and high triglycerides (>100 mg/dL in 0–9 years and >130 mg/dl in 10–19 years) in 4 (12.1%) patients. Elevated urinary albumin loss was found in 3 (9.1%) of the children.

**Table 1:** Clinical and biochemical parameters in children with tuberous sclerosis complex.

**Analyzed parameters**	**Children with TSC**
Number of patients	33
Age [years]	11.13 ± 4.03
Boys/girls	15/18 (45%/55%)
Positive family history	7 (21.2%)
BMI *Z-*score	0.31 ± 0.97
Arterial hypertension	7 (21.2%)
Developmental delay	22 (66.7%)
Epilepsy^*^	24 (72.7%)
Angiomyolipoma (*n*, %)	27 (81.8%)
Fat-poor angiomyolipoma (*n*, %)	9 (27.3%)
Angiomyolipoma [mm]	9.5 (6–25)
Renal cysts (*n*, %)	26 (78.8%)
Renal cysts [mm]	7.5 (5–10)
Antihypertensive medications	3 (9.1%)
Antiepileptic medications	17 (51.5%)
mTOR inhibitors	4 (12.1%)
eGFR ac. to Schwartz formula [ml/min/1.73m^2^]	130.8 ± 28.1
Cystatin *C* [ng/mL]	0.9 ± 0.2
Uric acid [mg/dL]	4.2 ± 0.9
Total cholesterol [mg/dL]	155 (135–169)
LDL cholesterol [mg/dL]	81.1 ± 28.6
HDL cholesterol [mg/dL]	57.5 ± 19.4
Triglycerides [mg/dL]	78.5 ± 28.7
Albuminuria [mg/24 h]	8.6 (4.6–14.6)

* *at present or in anamnesis*.

*TSC, tuberous sclerosis complex; BMI, body mass index; mTOR, mammalian target of rapamycin; eGFR, estimated glomerular filtration rate; LDL, low-density lipoprotein; HDL, high-density lipoprotein*.

### Blood Pressure and Arterial Damage in Children With Tuberous Sclerosis Complex

**Figure 3:**
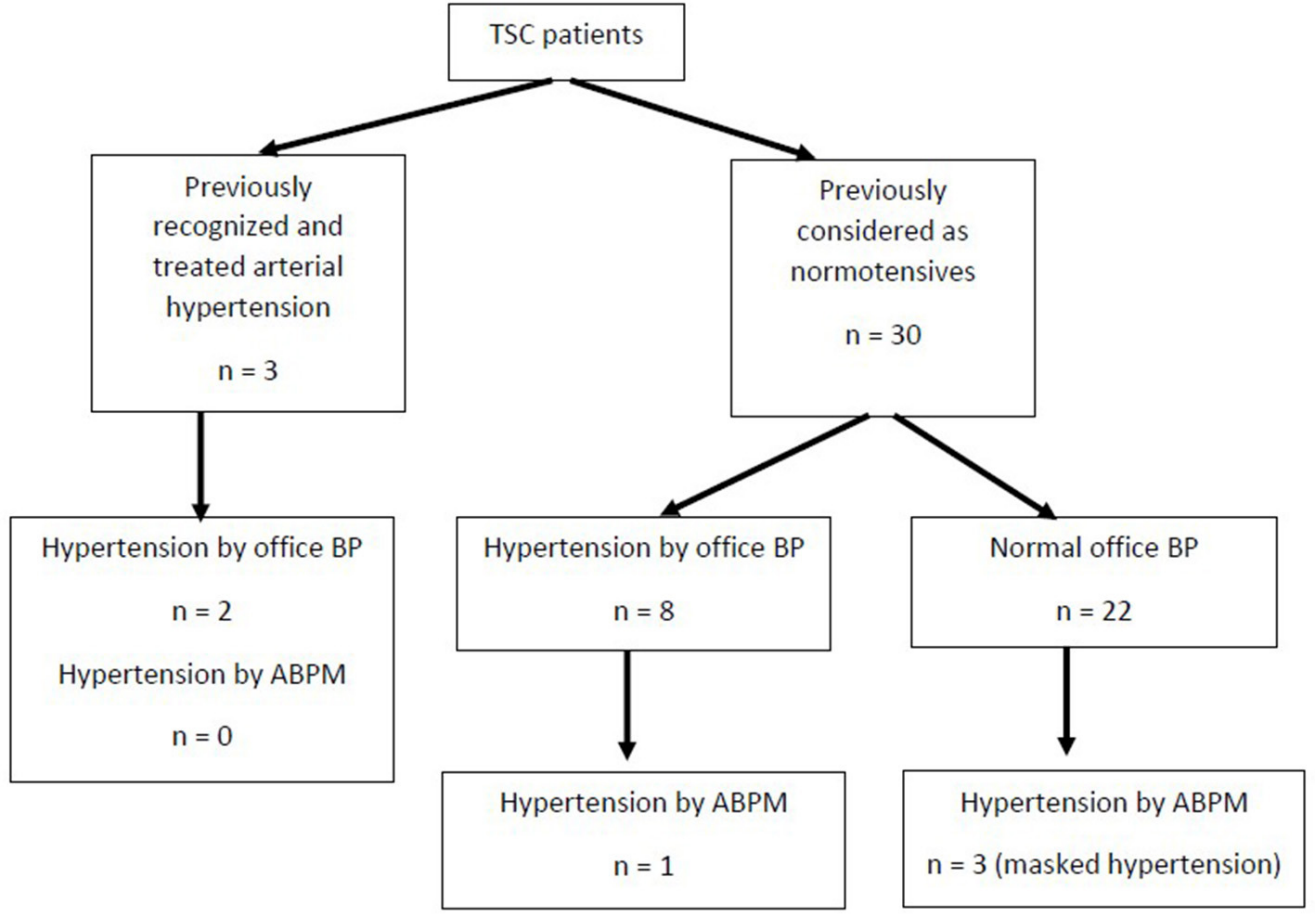
The results of blood pressure measurement in children with tuberous sclerosis complex (TSC, tuberous sclerosis complex; BP, blood pressure; ABPM, ambulatory blood pressure monitoring).

The comparison of blood pressure in children with TSC and healthy children was depicted in Table 2. There were no differences between the groups in terms of age, sex, and BMI *Z-*score. In the group of TSC patients, elevated (≥95th percentile) office systolic or diastolic blood pressure was revealed in 10 (30.3%) patients, including two patients with previously recognized arterial hypertension. Elevated blood pressure in ABPM was revealed in 4 (12.1%) patients—all of them were considered as normotensives before the study. Among these four patients, one 13-year-old girl also had elevated office blood pressure. Additionally, ABPM revealed masked hypertension in three patients. All three patients with previously recognized and treated arterial hypertension had normal ABPM results at the moment of the study. The analysis of blood pressure in TSC children was depicted in Figure 3. A disturbed circadian blood pressure profile was recognized in 16 TSC patients (including five patients with arterial hypertension). Children with TSC were characterized by significantly higher office peripheral and central blood pressure and 24-h ambulatory blood pressure than healthy peers. The groups did not differ significantly in 24-h pulse pressure, heart rate, and systolic and diastolic blood pressure dipping.

**Table 2:** Blood pressure in children with TSC and in healthy children.

**Parameter**	**Children with TSC**	**Control group**	***P***
Number of patients	33	33	–
Age [years]	11.13 ± 4.03	11.23 ± 3.28	
Boys/girls	15/18 (45%/55%)	15/18 (45%/55%)	1.000
BMI *Z-*score	0.31 ± 0.97	0.59 ± 1.24	0.170
**Office peripheral blood pressure**			
SBP [mm Hg]	115.6 ± 10.7	107.6 ± 8.7	0.001
SBP *Z-*score	0.82 ± 0.87	0.00 ± 0.50	<0.001
DBP [mm Hg]	69.1 ± 9.7	62.7 ± 7.3	0.004
DBP *Z-*score	0.9 ± 1.3	−0.02 ± 0.96	0.002
MAP [mm Hg]	85.6 ± 9.1	78.0 ± 6.9	<0.001
PP [mm Hg]	46.5 ± 6.7	44.9 ± 5.9	0.307
**Office central blood pressure**			
AoSBP [mm Hg]	98.6 ± 9.6	90.4 ± 6.9	<0.001
AoDBP [mm Hg]	71.2 ± 9.9	64.3 ± 7.3	0.002
AoMAP [mm Hg]	85.6 ± 9.1	78.1 ± 6.9	<0.001
AoPP [mm Hg]	27.4 ± 4.4	26.1 ± 4.0	0.227
**24-h ambulatory blood pressure**			
ABPM SBP 24 h [mm Hg]	114.5 ± 9.9	107.2 ± 5.2	<0.001
ABPM DBP 24 h [mm Hg]	65.5 ± 7.2	60.5 ± 3.9	<0.001
ABPM MAP 24 h [mm Hg]	81.9 ± 7.8	73.0 ± 4.7	<0.001
ABPM MAP 24 *Z-*score	−0.13 (−0.66 to 1.10)	−1.44 (−1.77 to −0.62)	<0.001
PP 24 h [mm Hg]	48.9 ± 5.4	46.7 ± 4.0	0.063
HR 24 h [bpm]	83 (73–91)	80 (75–86)	0.366
SBPL/24 h (%)	10 (2–25)	5 (2–9)	0.037
DBPL/24 h (%)	7 (2–15)	3 (1–4)	0.018
SBP DIP [%]	9.1 ± 5.2	11.2 ± 4.2	0.080
DBP DIP [%]	15.9 ± 7.5	17.4 ± 6.3	0.403

*TSC, tuberous sclerosis complex; BMI, body mass index; SBP, systolic blood pressure; DBP, diastolic blood pressure; MAP, mean arterial pressure; PP, pulse pressure; AoSBP, aortic systolic blood pressure; AoDBP, aortic diastolic blood pressure; AoMAP, aortic mean arterial pressure; AoPP, aortic pulse pressure; ABPM, ambulatory blood pressure monitoring; SBPL, systolic blood pressure load; DBPL, diastolic blood pressure load; DIP, dipping*.

**Figure 4:**
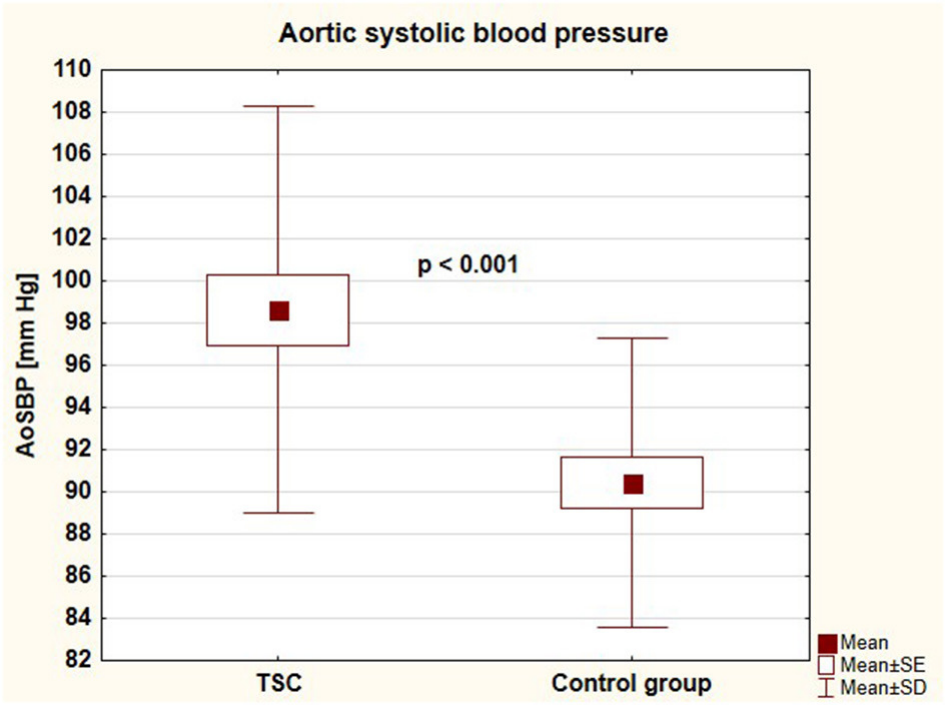
Aortic systolic blood pressure in children with tuberous sclerosis complex and in control group (AoSBP, aortic systolic blood pressure; TSC, tuberous sclerosis complex; SE, standard error; SD, standard deviation).

**Figure 5:**
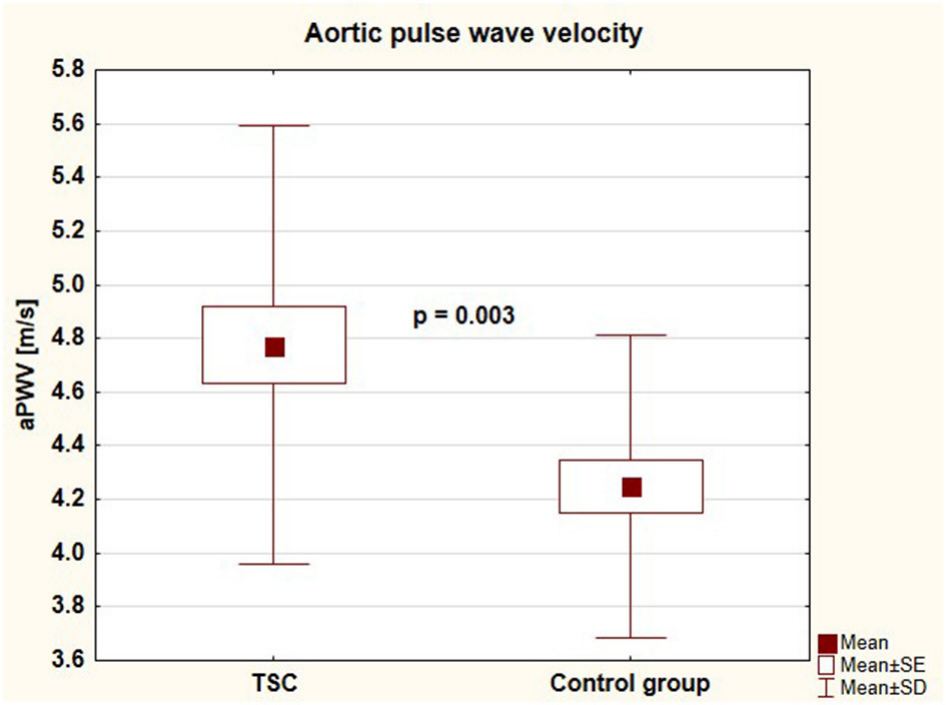
Aortic pulse wave velocity in children with tuberous sclerosis complex and in control group (aPWV, aortic pulse wave velocity; TSC, tuberous sclerosis complex; SE, standard error; SD, standard deviation).

**Figure 6:**
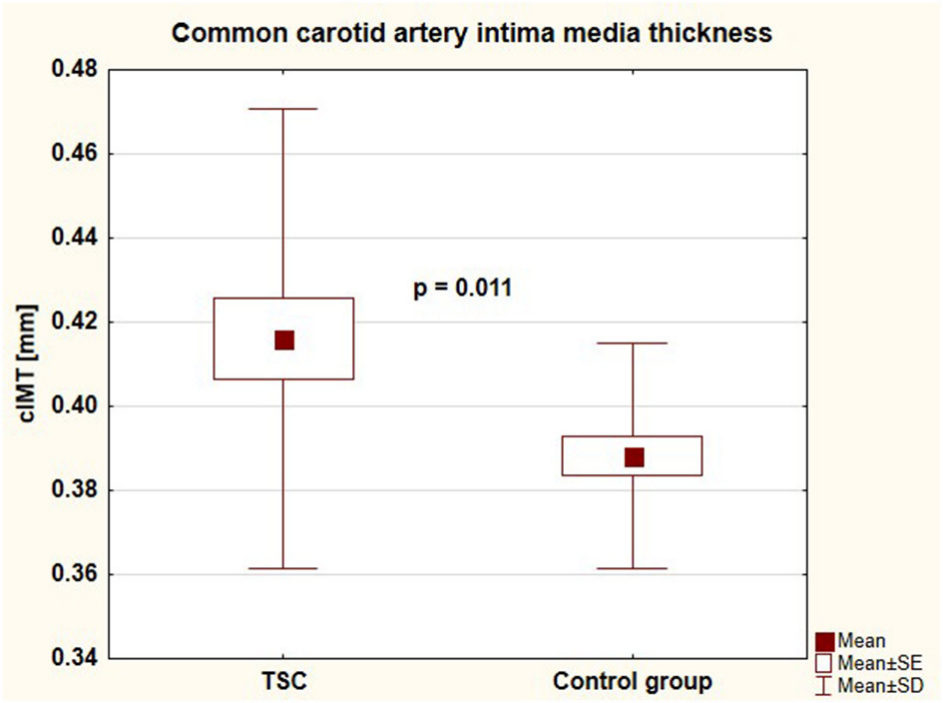
Common carotid artery intima media thickness in children with tuberous sclerosis complex and in control group (cIMT, common carotid artery intima media thickness; TSC, tuberous sclerosis complex; SE, standard error; SD, standard deviation).

Parameters of arterial damage in both groups were presented in Table 3. Patients with TSC were characterized by significantly faster aortic pulse wave velocity (4.76 ± 0.81 vs. 4.25 ± 0.56 [m/s], *p* = 0.003) and thicker common carotid artery intima-media thickness (0.42 ± 0.05 vs. 0.39 ± 0.03 [mm], *p* = 0.011) than healthy individuals. There were no differences between the groups regarding augmentation index, subendocardial viability ratio, local carotid arterial dimension, or stiffness parameters. Central systolic blood pressure, aortic pulse wave velocity, and common carotid intima-media thickness in both groups were presented in Figures 4–6, respectively.

**Table 3:** Parameters of arterial damage (early vascular aging) in children with tuberous sclerosis complex and in healthy children.

**Parameter**	**Children with TSC**	**Control group**	***P***
aPWV	4.76 ± 0.81	4.25 ± 0.56	0.003
aPWV *Z-*score	−0.14 ± 1.15	−0.96 ± 0.87	0.002
AIx75HR [%]	8.71 ± 15.90	5.24 ± 11.12	0.319
Buckberg SEVR [%]	135.3 (120–159)	145.8 (130–169)	0.115
cIMT [mm]	0.42 ± 0.05	0.39 ± 0.03	0.011
cIMT *Z-*score	0.81 ± 1.21	0.21 ± 0.55	0.007
ET beta	3.4 (2.4–4.1)	3.7 (2.7–4.6)	0.510
ET Ep [kPa]	36 (28–51)	41 (31–50)	0.748
ET AC [mm^2^/kPa]	1.14 (0.91–1.52)	1.02 (0.82–1.29)	0.346
ET AIx [%]	0.00 (−6.90 to 17.60)	−3.29 (−5.10 to 0.00)	0.072
ET PWVbeta [m/s]	3.60 (3.20–4.30)	3.75 (3.30–4.20)	0.944
ET D max [mm]	5.85 (5.24–6.16)	5.83 (5.06–6.38)	0.517
ET D min [mm]	4.78 (4.50–5.41)	4.95 (4.32–5.54)	0.807
ET DATmax [ms]	140.00 (123.00–178.00)	128.00 (124.00–142.00)	0.146

*aPWV, aortic pulse wave velocity; AIx75HR, augmentation index normalized to heart rate of 75 beats per minute; SEVR, subendocardial viability ratio; cIMT, common carotid artery intima-media thickness; ET, ECHO-tracking; beta, stiffness index; Ep, pressure strain elasticity modulus; AC, arterial compliance; AIx, augmentation index; D max, maximal diameter of right common carotid artery; D min, minimal diameter of right common carotid artery; DATmax, acceleration time to common carotid artery maximal diameter*.

When we excluded 7 children with arterial hypertension from the study group, we found that remaining 26 children with TSC were still characterized by significantly faster aortic pulse wave velocity (aPWV: 4.73 ± 0.83 vs. 4.25 ± 0.56 [m/s], *p* = 0.011, aPWV *Z-*score: −0.18 ± 1.18 vs. −0.96 ± 0.87, *p* = 0.006) and thicker common carotid artery intima media thickness (cIMT: 0.41 ± 0.05 vs. 0.39 ± 0.03 [mm], *p* = 0.042). The groups still did not differed in age (10.93 ± 4.07 vs. 11.23 ± 3.28 [years], *p* = 0.754) and sex (boys/girls-−11/15 vs. 15/18, *p* = 0.809).

### The Determinants of Blood Pressure and Arterial Parameters in Children With Tuberous Sclerosis Complex

In TSC patients, aortic systolic blood pressure and peripheral office diastolic blood pressure *Z-*score correlated significantly with maximal diameter of the renal cyst (*R* = 0.419, *p* = 0.033 and *R* = 0.484, *p* = 0.012, respectively). Aortic systolic blood pressure and mean arterial pressure during 24 h *Z-*score correlated with serum cystatin C concentration (*r* = 0.377, *p* = 0.030 and *R* = 0.433, *p* = 0.013, respectively).

Boys with TSC did not differ from girls in terms of aortic pulse wave velocity (aPWV [m/s]: 4.85 ± 0.84 vs. 4.72 ± 0.81 [m/s], *p* = 0.660; aPWV *Z-*score:−0.07 ± 1.12 vs.−0.21 ± 1,21, *p* = 0.752) or common carotid artery intima media thickness (cIMT [mm]: 0.43 ± 0.06 vs. 0.41 ± 0.05, *p* = 0.383; cIMT *Z-*score: 0.87 ± 1.14 vs. 0.75 ± 1.30, *p* = 0.797). We found a positive correlation between aPWV *Z-*score and daily urinary albumin loss (*R* = 0.412, *p* = 0.029) and trend toward a positive correlation between aPWV *Z-*score and maximal AML diameter (*R* = 0.339, *p* = 0.058).

As for parameters of local carotid artery stiffness in patients with TSC, pressure strain elasticity modulus (Ep) correlated with mean arterial pressure during 24 h *Z-*score (MAP 24 h *Z-*score) (*R* = 0.409, *p* = 0.020), arterial compliance (AC) correlated with systolic blood pressure load during 24 h (*R* = −0.367, *p* = 0.036) and with MAP 24 h *Z-*score (*R* = −0.538, *p* = 0.001) and local pulse wave velocity (PWVbeta) also correlated with MAP 24 h *Z-*score (*R* = 0.411, *p* = 0.019).

## Discussion

This is the first study to analyze central blood pressure and the phenomenon of early vascular aging in pediatric patients with tuberous sclerosis complex. We have compared our results with perfectly sex- and age-matched healthy peers. Our analysis revealed that TSC patients were characterized by significantly higher central systolic blood pressure, aortic pulse wave velocity, and common carotid artery intima-media thickness. We have found that blood pressure correlated with serum cystatin C and renal cyst diameter in this group of patients. There was also positive relation between pulse wave velocity and the extent of renal involvement. Parameters of local artery stiffness correlated with ambulatory blood pressure.

Our analysis has involved 33 children with already diagnosed TSC hospitalized in one tertiary center of pediatric nephrology. Our center has developed a program of nephrological care for pediatric TSC patients in 2017 based on ITSCCG and the Polish Society of Nephrology recommendations (16, 29). The preliminary analysis of our TSC cohort has already been published in a local medical journal (30). In addition, we aimed to analyze blood pressure and vascular phenotype in these patients. The clinical characteristics of our cohort do not differ from those of patients with TSC in large international registries including TOSCA registry (TuberOus SClerosis registry to increase disease Awareness) (3).

Renal lesions including AMLs were more frequently found in our group (90.9% of children) than in other pediatric patients groups—renal involvement is estimated to occur in approximately 38.5−55% of preschool children and 75–80% of schoolchildren (13, 20, 31, 32). The higher incidence of renal manifestations in our group results from the specificity of patients referred to our Department—the main indication was the presence of focal renal lesions found on imaging studies. In the analysis of patients from the TOSCA registry, AML lesions were present in 51.8% of patients (13). Renal cysts in most patients with TSC are small and localized in the subcortical region. Patients with simultaneous deletion of *TSC2* and *PKD1* genes are characterized by a very severe phenotype with large cysts from early childhood. Many of these patients develop a rapid progression to end-stage renal disease already in the second or third decade of life (13, 32). In our study group, renal cysts were found in three-quarters of patients. The largest cysts were found in two boys with a genetically confirmed mutation in the *PKD*1 gene. Of note, in our group of patients, hyperfiltration was found in as many as one-third of the subjects. This frequency is even higher than in the study of Belgian authors (20). It has been postulated that hyperfiltration in patients with TSC is caused by overactivation of the mTOR pathway in the glomerulus and, as in diabetic nephropathy, may be a risk factor for progression of renal disease.

In the analyzed group of patients with TSC, hypertension was present in 21.2% of patients, which is higher than the average prevalence of hypertension in the population of healthy children (3–5%) (33) and is also higher compared to the percentage of patients with hypertension in the Belgian study (13.0%) (20). In adults with TSC, arterial pressure has been shown to depend on the size and number of both AML lesions and cysts (32, 34). Severe hypertension in this group of patients may also be related to the presence of vascular lesions—renal artery stenosis sometimes accompanied by mid-aortic syndrome (MAS) (10). The ITSCCG recommendations clearly state the need for regular blood pressure measurements in this group of patients (1). The idea of measurement of central blood pressure has gained much attraction in recent years (35). Also, pediatric data indicate that central systolic blood pressure is a significant predictor of target organ damage, as valuable as 24-h peripheral blood pressure monitoring (36). We found that pediatric patients with TSC were characterized by a significantly higher central systolic blood pressure than healthy peers. Cystatin C and renal cyst diameter were significant determinants of elevated blood pressure in our cohort. The latter finding is consistent with the results of a multicenter study in children with autosomal dominant polycystic kidney disease (37) and adult TSC registries (32, 34).

Arterial damage is one of the first alterations observed in pediatric populations with increased cardiovascular risk, such as chronic kidney disease (CKD) (38), primary hypertension (PH) (39), diabetes mellitus (DM) type 1 (40), or familial hypercholesterolemia (41). Elastic properties of arteries in high-risk pediatric patients are similar to vascular changes in the elderly and are defined as early vascular aging. The concept of EVA is based on findings that individuals with PH, DM or CKD present with more advanced signs of arterial aging than their healthy peers (15, 42). Among different indices of EVA, evaluation of common carotid artery intima-media thickness and aortic (carotid-femoral) pulse wave velocity are considered as the most valuable ones with generally accepted pediatric normative values (27, 28, 43) and well-established association with hard-end points in the adult population (44, 45).

Our study is the first to reveal signs of EVA in pediatric patients with TSC. The primary cause of EVA in this group of patients is yet to be solved. Nevertheless, there is some possible explanation for this result. Firstly, vascular senescence in this population could be the effect of blood pressure rise as blood pressure is the primary determinant of cIMT and aPWV in the general pediatric population (27, 43). Moreover, initial functional and morphological vascular changes can be regarded as adaptive in response to increased blood pressure (42). Indeed, office peripheral and central blood pressure was significantly higher in this group of TSC patients compared to healthy age- and sex-matched peers. Nevertheless, blood pressure remained within normal limits in most of these patients, and arterial hypertension was found in approximately 20% of the studied children. In addition, no significant correlation between blood pressure and parameters of arterial damage except for carotid artery stiffness has been revealed in our patients. Also, cIMT and aPWV were still elevated in TSC patients after the exclusion of 7 hypertensive children. Metabolic factors do not seem to play a role either, as these children generally had normal lipid parameters and did not differ in BMI from the control group. Thus, it is possible that other factors might be to blame.

Vascular anomalies have been described in TSC patients for decades (9, 10), including renal artery stenosis (sometimes with the mid-aortic syndrome) and aortal aneurysms also in small children. The youngest reported TSC case is an infant who died of aortic aneurysm rupture at the age of 4.5 months (46). Vascular smooth muscle cells (SMCs) are not terminally differentiated and can transform to proliferating and migrating cells with loss of contractile protein expression and increased synthesis of extracellular matrix proteins. In SMCs, mTOR signaling was found to influence cell differentiation. Cao et al. revealed in an experimental model that the activation of the mTOR pathway with *Tsc2* deficiency leads to SMC proliferation and de-differentiation *in vitro* and *in vivo*, which can be reversed with rapamycin treatment (8). Recently, a disruption of the *Tsc1* gene was also found to induce a degradative smooth muscle cell phenotype (47). The authors hypothesize that the proliferation of degradative SMCs within the media causes arterial dysfunction in TSC patients. A histological study of a thoracoabdominal aneurysm in a child with TSC revealed SMC hyperplasia in the inner media with diminished actin expression and extensive fragmentation of elastic fibers (8). Our preliminary results suggest that the proliferation and degradation of vascular SMCs are already present in children with TSC without macroscopically evident arterial dilations or stenoses.

In addition, the extent of renal involvement might play a role in the development of blood pressure rise and arterial damage as blood pressure was associated with cystatin C and cyst diameter and aortic pulse wave velocity with urine albumin loss and AML diameter. A positive correlation between albuminuria and arterial stiffness has been revealed in adults with CKD (48) and DM 2 (49) and in the general young adult population from the Malmö offspring study (50). Albuminuria is not only a marker of renal damage but is a well-established indicator of endothelial dysfunction, which may result in an altered arterial wall and increased arterial stiffness. Gil-Ortega et al. found that increased albuminuria was associated with abnormalities in arterial wall structure (elastin loss) in an experimental rat model (51). A similar association might also be seen in TSC patients. Inversely, increased urinary albumin loss could be the result of the downstream transmission of pressure pulsatility to the level of renal microcirculation (52).

TSC is one of those rare entities for which there is a targeted treatment that directly affects the mechanism of the disease. Numerous data from both single case reports and multicenter clinical trials indicate high efficacy of mTOR inhibitors (everolimus and sirolimus/rapamycin) in the treatment of virtually all symptoms of the disease, including central nervous system lesions (53), renal AML (54) or lung lesions (55). The timing of initiation of mTOR inhibitors in TSC patients is a matter of debate. Recent multicenter studies have proven the safety and efficacy of mTOR inhibition in TSC patients even younger than 2 years (56, 57). Of note, *in vitro* studies showed that the mTOR inhibitor rapamycin promotes SMC differentiation toward a contractile phenotype (58). Inhibition of the mTOR signaling pathway has been used for many years in medicine in rapamycin-diluting stents that prevent SMC proliferation and artery occlusion after percutaneous interventions (59). There is already some evidence indicating that early treatment in high-risk children and adolescents [e.g., in patients with CKD (60) or PH (61)] could reverse EVA and possibly prevent premature cardiovascular events. Whether mTOR inhibitor treatment or any other medical measures (e.g., antihypertensive treatment) would prevent or even reverse negative alterations in arteries of TSC patients is still unknown and should certainly be investigated in years to come.

Some limitations to our research need to be listed. Firstly, the study's cross-sectional nature precludes drawing final conclusions on the relation between blood pressure, arterial damage, and clinical parameters in the studied TSC patients (e.g., the link between aPWV and albuminuria). Secondly, the study population was small and heterogeneous in terms of age, presence of arterial hypertension, the extent of renal involvement, genetic background (*TSC1, TSC2, TSC2*+*PKD1*), and treatment with mTOR inhibitors. As arterial hypertension was recognized previously in only 3 patients, we could not analyze the influence of duration of hypertension on arterial damage in TSC patients.

## Conclusions

Children with tuberous sclerosis complex are at risk of elevated central blood pressure and early vascular aging. In this group of patients, early vascular aging might be caused by uncontrolled activation of the mTOR signaling pathway in vascular smooth muscle cells. Additionally, in children with TSC, blood pressure and arterial stiffness are related to renal involvement. There is a need for studies on possible vasoprotective measures in TSC patients, including the use of mTOR inhibitors.

## Data Availability Statement

The raw data supporting the conclusions of this article will be made available by the authors, without undue reservation.

## Ethics Statement

The studies involving human participants were reviewed and approved by Bioethics Committee, Medical University of Warsaw (approval no. KB/145/2017, 4th July 2017). Written informed consent to participate in this study was provided by the participants' legal guardian/next of kin and the participants (≥16 years).

## Author Contributions

PS, AMW, and MS drafted and revised the manuscript. PS, SJ, MB, and MP-T contributed to the conception and design of the work. PS, AMW, MS, MB, AJ-K, and PB contributed to the acquisition of data. PS, AMW, MS, SJ, MB, AJ-K, PB, and MP-T contributed to the analysis or interpretation of the data. SJ and MP-T critically revised the manuscript. All authors gave their final approval and agreed to be accountable for all aspects of this work ensuring its integrity and accuracy.

## Funding

This research was funded from the statutory funds of the Department of Pediatrics and Nephrology, Medical University of Warsaw.

## Conflict of Interest

The authors declare that the research was conducted in the absence of any commercial or financial relationships that could be construed as a potential conflict of interest.

## Publisher's Note

All claims expressed in this article are solely those of the authors and do not necessarily represent those of their affiliated organizations, or those of the publisher, the editors and the reviewers. Any product that may be evaluated in this article, or claim that may be made by its manufacturer, is not guaranteed or endorsed by the publisher.
